# High cholesterol and low triglycerides are associated with total lumbar bone mineral density among adults aged 50 years and over: The NHANES 2017–2020

**DOI:** 10.3389/fmed.2022.923730

**Published:** 2022-08-08

**Authors:** Peng Wang, Cong Chen, Chunhao Song, Jun Jia, Yuanhao Wang, Weidong Mu

**Affiliations:** ^1^Department of Spine Surgery, Weihai Municipal Hospital, Cheeloo College of Medicine, Shandong University, Weihai, China; ^2^Department of Medical Imaging, Weihai Wendeng District People’s Hospital, Weihai, China; ^3^Department of Traumatic Orthopaedics, Shandong Provincial Hospital Affiliated to Shandong First Medical University, Jinan, China

**Keywords:** total cholesterol, triglycerides, lumbar bone mineral density, osteoporosis, NHANES

## Abstract

**Background:**

The association between cholesterol and triglycerides with the lumbar bone mineral density (BMD) was widely investigated, but the results remained conflicting. This study aimed to investigate the relationship between total cholesterol, triglycerides, and total lumbar BMD in adults.

**Materials and methods:**

This cross-sectional study included 1,985 individuals aged 50 years and over. The data on total cholesterol, triglycerides, total lumbar BMD, and other covariates were obtained from the National Health and Nutritional (NHANES) between 2017 and March 2020 pre-pandemic. Multivariate logistic regression models were utilized to investigate the association between cholesterol, triglycerides, and total lumbar BMD. Smooth curve fittings and generalized additive models were also used to analyze the potential non-linearity.

**Results:**

A total of 901 men and 1,084 women with a mean age of 63.02 ± 8.72 years (age 50–80 years) were included in this study. In multivariate regression analysis, the association between cholesterol and total lumbar BMD was negative (β = −0.026, 95% *CI*: −0.033, −0.020). This relationship still existed after adjusted for gender and race (β = −0.018, 95% *CI*: −0.025, −0.012) and fully adjusted for all covariates (β = −0.022, 95% *CI*: −0.029, −0.015). The association between triglycerides and total lumbar BMD was positive (β = 0.024, 95% *CI*: 0.017, 0.031). This relationship still existed after adjusted for gender and race (β = 0.021, 95% *CI*: 0.015, 0.028) and fully adjusted for all covariates (β = 0.021, 95% *CI*: 0.014, 0.028). In threshold effect analysis, the relationship between triglycerides and total lumbar BMD was an inverted *U*-shaped curve with the inflection point at 2.597 mmol/L.

**Conclusion:**

High levels of total cholesterol and relatively low levels of triglycerides are significantly associated with the total lumbar BMD in adults aged 50 years and over.

## Introduction

Osteoporosis is one of the most prevalent public health threats in elderly populations, characterized by microarchitectural deterioration, reduced lumbar bone mineral density (BMD), and skeletal fragility. The diagnosis of osteoporosis is based on the BMD measured by dual-energy X-ray absorptiometry (DXA). Fracture is the most serious clinical consequence of osteoporosis. It is estimated that 1.5 million fractures were caused by osteoporosis per year in the United States (1). Osteoporosis-related fractures can result in poor quality of life, high healthcare costs, and an increased risk of death. Low BMD is one of the important risk factors for fractures (2).

Atherosclerosis is another severe hazard to health and quality of life. The metabolic disorder of cholesterol and triglycerides is the main cause of atherosclerosis. Atherosclerosis and osteoporosis often coincide with each other in elderly people (3), yielding the metabolism of cholesterol and triglycerides associated with BMD. To assess the potential role of cholesterol and triglycerides in osteoporosis, different studies have been investigated but the results remained conflicting. Therefore, the objective of the current study was to evaluate the relationship between total cholesterol, triglycerides, and total lumbar BMD in adults aged over 50 years using a nationally representative sample from the NHANES 2017–2020.

## Materials and methods

### Study population

Data were obtained from the NHANES between 2017 and March 2020 pre-pandemic for this study. NHANES databases are cross-sectional surveys conducted by the National Center for Health Statistics, providing multitudinous health and nutrition data of the general United States population.

The data of NHANES are publicly available on the internet. Full detailed information about NHANES can be found on the website.^1^ The conduct of NHANES was approved by the NCHS Ethics Review Board, and the signed written informed consents were obtained from all included participants (4).

**FIGURE 1:**
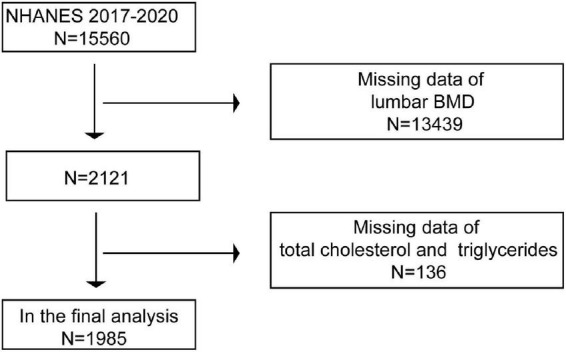
Flow chart of sample selection from NHANES 2017–2020.

A total of 15,560 individuals aged 50 years and over were enrolled for this study from the NHANES between 2017 and March 2020 pre-pandemic. After exclusion of 13,575 subjects with missing total lumbar BMD (*n* = 13439) or total cholesterol, triglycerides (*n* = 136) data, and 1,985 subjects remained for the final analysis (Figure 1).

### Study variables

The exposure variables were total cholesterol and triglycerides. Total cholesterol and triglycerides were conducted with blood serum at Advanced Research and Diagnostic Laboratory, using Roche Cobas 6000 (c501 module).

The outcome variable was total lumbar BMD. The measurement of total lumbar BMD was provided by DAX scans, administered by trained and certified radiology technologists. The spine scans were acquired on Hologic Discovery model A densitometers (Hologic, Inc., Bedford, MA, United States) in 2017–2018 and on Hologic Horizon model A densitometers (Hologic, Inc., Bedford, MA, United States) in 2019–2020. A cross-calibration study was conducted to assure the accuracy of the NHANES longitudinal assessment.

Multivariate models contain variables that might confound the links between total cholesterol, triglycerides, and total lumbar BMD. The data on gender, age, race, and marital status were obtained from questionnaires. The data on alanine aminotransferase, albumin, alkaline phosphatase, bicarbonate, blood urea nitrogen, chloride, creatinine, globulin, glucose, gamma-glutamyl transferase, iron, osmolality, phosphorus, sodium, total bilirubin, total calcium, total protein, and uric acid were obtained from standard biochemistry profile analysis with a Roche Cobas 6000. Detailed information about these covariates can be obtained from the NHANES website.

### Statistical analyses

The study participants were stratified into quartiles according to total cholesterol and triglycerides levels. Weighted multivariable linear regression models were applied to analyze the association between total cholesterol, triglycerides, and total lumbar BMD. Weighted smooth curve fittings and generalized additive models were used to analyze the potential non-linearity. Two-piecewise linear regression models were used to calculate the threshold effects if non-linearity associations existed. Three models were built: Model I, an unadjusted model; Model II, minimally adjusted for gender, age, and race; Model III, fully adjusted for all covariates (listed in Tables 1, 2). All analyses were performed using the Empower-Stats software (version 2.0. X&Y Solutions, Boston, MA, United States) and statistical software R (version 3.4.3). The *p* < 0.05 was considered statistically significant.

**TABLE 1:** Weighted characteristics of study population based on total cholesterol levels quartiles.

CHO levels (mmol/L)	Q1	Q2	Q3	Q4	*P*-value
Age (years)	64.19 ± 8.97	62.01 ± 8.46	61.91 ± 8.40	61.49 ± 8.45	<0.001
Gender (%)					<0.001
Male	63.52	48.15	33.89	33.93	
Female	36.48	51.85	66.11	66.07	
RACE (%)					0.715
1	69.67	65.19	69.67	65.97	
2	10.65	9.88	9.13	11.07	
3	5.13	6.37	4.76	6.00	
4	14.55	18.56	16.44	16.97	
MS (%)					0.005
1	69.15	59.54	69.10	61.76	
2	30.64	40.46	30.90	38.10	
3	0.22			0.15	
ALT (IU/L)	23.82 ± 15.79	23.59 ± 22.58	21.06 ± 11.19	21.72 ± 14.32	0.017
AL (g/dL)	40.18 ± 3.21	40.48 ± 2.96	40.69 ± 2.66	40.95 ± 2.82	0.001
ALP (IU/L)	81.29 ± 29.16	79.18 ± 26.63	79.42 ± 21.83	81.78 ± 24.19	0.269
BC (mmol/L)	25.71 ± 2.61	26.17 ± 2.61	25.83 ± 2.23	25.69 ± 2.38	0.006
BUN (mmol/L)	6.14 ± 2.23	5.82 ± 1.85	5.69 ± 1.45	5.71 ± 1.92	<0.001
CL (mmol/L)	101.58 ± 3.45	101.33 ± 2.73	100.84 ± 2.57	100.45 ± 2.89	<0.001
CR (umol/L)	84.95 ± 47.38	80.81 ± 37.07	76.17 ± 18.35	77.13 ± 33.81	<0.001
GLO (g/L)	29.45 ± 4.86	29.45 ± 4.45	29.77 ± 4.31	30.13 ± 3.75	0.043
GlU (mmol/L)	6.78 ± 2.930	5.62 ± 1.67	5.57 ± 1.67	5.63 ± 2.08	<0.001
GGT (IU/L)	35.05 ± 47.23	34.21 ± 47.76	28.74 ± 30.72	36.03 ± 42.28	0.029
Iron (umol/L)	15.78 ± 6.21	16.31 ± 5.86	15.99 ± 5.44	16.14 ± 5.44	0.522
OSM (mmol/Kg)	283.84 ± 6.89	281.91 ± 5.13	281.21 ± 4.83	280.63 ± 5.60	<0.001
PHO (mmol/L)	1.14 ± 0.18	1.13 ± 0.16	1.18 ± 0.16	1.18 ± 0.16	<0.001
SOD (mmol/L)	140.80 ± 3.27	140.57 ± 2.54	140.31 ± 2.38	139.94 ± 2.73	<0.001
BIL (umol/L)	9.03 ± 5.15	8.69 ± 4.84	7.77 ± 3.89	7.79 ± 3.94	<0.001
CAL (mmol/L)	2.31 ± 0.10	2.32 ± 0.09	2.34 ± 0.09	2.34 ± 0.09	<0.001
PRO (g/L)	69.64 ± 4.81	69.94 ± 4.56	70.46 ± 4.40	71.08 ± 3.84	<0.001
TG (mmol/L)	1.48 ± 0.80	1.40 ± 0.72	1.50 ± 0.81	1.99 ± 1.53	<0.001
UA (umol/L)	5.380 ± 1.41	5.36 ± 1.34	5.26 ± 1.26	5.40 ± 1.43	0.392
BMD (gm/cm^2^)	1.05 ± 0.16	1.02 ± 0.18	0.98 ± 0.15	0.99 ± 0.17	<0.001

Mean + SD for continuous variable;% for Categorical variable; *P*-value was calculated by weighted chi-square test.

CHO, cholesterol; MS, marital status; ALT, alanine aminotransferase; AL, albumin; ALP, alkaline phosphatase; BC, bicarbonate; BUN, blood urea nitrogen; CL, chloride; CR, creatinine; GLO, globulin; GLU, glucose; GGT, gamma glutamyl transferase; OSM, osmolality; PHO, phosphorus; SOD, sodium; BIL, total bilirubin; CAL, total calcium; PRO, total protein; TG, triglycerides; UA, uric acid; BMD, total spine BMD.

RACE: 1, White Race; 2, Black Race; 3, Mexican Race; 4, Other Race.

MS: 1, Living with Partner; 2, Separated; 3, Don’t Know.

**TABLE 2:** Weighted characteristics of study population based on triglycerides levels quartiles.

TG levels (mmol/L)	Q1	Q2	Q3	Q4	*P*-value
Age (years)	61.89 ± 8.64	62.84 ± 8.35	62.48 ± 8.57	62.22 ± 8.92	0.3639
Gender (%)					<0.001
Male	44.62	42.24	38.58	53.05	
Female	55.38	57.76	61.42	46.95	
RACE (%)					<0.001
1	65.78	67.68	69.95	66.79	
2	15.47	11.53	8.38	5.40	
3	3.19	5.55	5.86	7.60	
4	15.55	15.24	15.81	20.21	
MS (%)					0.002
1	59.64	60.89	69.24	69.42	
2	40.06	39.04	30.76	30.58	
3	0.29	0.07			
ALT (IU/L)	20.84 ± 13.31	22.24 ± 22.98	22.47 ± 13.11	24.52 ± 14.17	0.0070
AL (g/dL)	40.63 ± 2.90	40.64 ± 2.96	40.37 ± 2.90	40.69 ± 2.93	0.3067
ALP (IU/L)	79.16 ± 29.26	79.35 ± 22.63	81.41 ± 27.23	81.59 ± 22.36	0.2787
BC (mmol/L)	25.99 ± 2.37	26.00 ± 2.42	25.81 ± 2.33	25.61 ± 2.72	0.0482
BUN (mmol/L)	5.76 ± 1.82	5.70 ± 1.74	5.83 ± 1.88	6.07 ± 2.05	0.0100
CL (mmol/L)	101.38 ± 2.46	101.14 ± 3.12	101.40 ± 2.93	100.22 ± 3.07	<0.0001
CR (umol/L)	79.36 ± 39.14	77.88 ± 31.49	80.53 ± 38.83	80.90 ± 31.92	0.5286
GLO (g/L)	29.36 ± 4.61	29.72 ± 4.63	29.62 ± 4.28	30.13 ± 3.84	0.0539
GLU (mmol/L)	5.58 ± 1.55	5.52 ± 1.21	5.70 ± 1.79	6.74 ± 3.36	<0.0001
GGT (IU/L)	26.66 ± 27.57	29.30 ± 32.79	34.37 ± 48.93	43.48 ± 53.10	<0.0001
Iron (umol/L)	15.30 ± 5.04	17.09 ± 6.21	15.87 ± 5.97	15.92 ± 5.46	<0.0001
OSM (mmol/Kg)	281.30 ± 5.02	281.57 ± 5.64	282.52 ± 5.83	282.02 ± 6.37	0.0046
PHO (mmol/L)	1.15 ± 0.16	1.15 ± 0.17	1.17 ± 0.16	1.16 ± 0.16	0.2722
SOD (mmol/L)	140.30 ± 2.44	140.51 ± 2.75	140.86 ± 2.86	139.89 ± 2.85	<0.0001
BIL (umol/L)	8.15 ± 4.36	9.19 ± 5.14	7.83 ± 3.75	8.04 ± 4.53	<0.0001
CAL (mmol/L)	2.31 ± 0.08	2.33 ± 0.10	2.32 ± 0.09	2.34 ± 0.10	<0.0001
CHO (mmol/L)	4.68 ± 0.92	4.97 ± 1.09	5.09 ± 0.98	5.31 ± 1.24	<0.0001
PRO (g/L)	69.99 ± 4.67	70.36 ± 4.43	70.00 ± 4.52	70.81 ± 4.09	0.0102
UA (umol/L)	4.80 ± 1.20	5.29 ± 1.30	5.57 ± 1.31	5.72 ± 1.44	<0.0001
BMD (gm/cm^2^)	0.97 ± 0.17	1.00 ± 0.17	1.01 ± 0.15	1.06 ± 0.17	<0.0001

Mean + SD for continuous variable;% for Categorical variable; *P*-value was calculated by weighted chi-square test.

TG, triglycerides; MS, marital status; ALT, alanine aminotransferase; AL, albumin; ALP, alkaline phosphatase; BC, bicarbonate; BUN, blood urea nitrogen; CL, chloride; CR, creatinine; GLO, globulin; GLU, glucose; GGT, gamma glutamyl transferase; OSM, osmolality; PHO, phosphorus; SOD, sodium; BIL, total bilirubin; CAL, total calcium; CHO, cholesterol; PRO, total protein; UA, uric acid; BMD, total spine BMD.

RACE: 1, White Race; 2, Black Race; 3, Mexican Race; 4, Other Race.

MS: 1, Living with Partner; 2, Separated; 3, Don’t Know.

## Results

There are 901 men and 1,084 women included in this study. Age averaged 63.02 ± 8.72 years with a range of 50–80 years. Baseline characteristics of all subjects, classified by quartiles of total cholesterol and triglycerides levels, are, respectively, presented in Tables 1, 2. As shown in Table 1, subjects with lower total cholesterol levels were older and the lowest lumbar BMD was in the Q3 group. In Table 2, subjects with lower triglycerides levels had lower BMD and the lowest lumbar BMD was in the Q1 group.

The association between total cholesterol and total lumbar BMD was negative in all three regression models (Table 3): model 1 (β = −0.026, 95% *CI*: −0.033, −0.020); model II (β = −0.018, 95% *CI*: −0.025, −0.012); model III (β = −0.022, 95% *CI*: −0.029, −0.015). Stratified by quartile level of total cholesterol, the trend test remained significant in Q3 and Q4 subgroups (*p* < 0.001). In the subgroup analysis stratified by gender, this negative association remained in the women group: model 1 (β = −0.027, 95% *CI*: −0.036, −0.017); model II (β = −0.028, 95% *CI*: −0.037, −0.019); model III (β = −0.028, 95% *CI*: −0.036, −0.019). In the men group, this association was no longer significant in models I and II but became negative after controlling in the fully adjusted model III (β = −0.011, 95% *CI*: −0.022, −0.001).

**TABLE 3:** Associations between total cholesterol (mmol/L) and total spine bone mineral density (BMD) (gm/cm^2^).

	Model I OR (95% CI) *P*	Model II OR (95% CI) *P*	Model III OR (95% CI) *P*
CHO	−0.026 (−0.033, −0.020) <0.001	−0.018 (−0.025, −0.012) <0.001	−0.022 (−0.029, −0.015) <0.001
**CHO (Quartile)**			
Q1	Reference	Reference	Reference
Q2	−0.028 (−0.049, −0.007) 0.008	−0.015 (−0.035, 0.005) 0.141	−0.009 (−0.028, 0.011) 0.375
Q3	−0.070 (−0.090, −0.049) <0.001	−0.045 (−0.066, −0.025) 0.001	−0.039 (−0.059, −0.019) <0.001
Q4	−0.060 (−0.081, −0.039) <0.001	−0.037 (−0.057, −0.016) <0.001	−0.041 (−0.062, −0.020) <0.001
*P* for trend	<0.001	<0.001	<0.001
**Gender**			
Males	−0.006 (−0.016, 0.003) 0.181	−0.005 (−0.014, 0.005) 0.347	−0.011 (−0.022, −0.001) 0.029
Females	−0.027 (−0.036, −0.017) <0.001	−0.028 (−0.037, −0.019) <0.001	−0.028 (−0.036, −0.019) <0.001

Model I: None covariates were adjusted; Model II: gender (not adjusted for in the subgroup analyses), age and race were adjusted; Model III: gender (not adjusted for in the subgroup analyses), age, race, marital status, alanine aminotransferase, albumin, alkaline phosphatase, bicarbonate, blood urea nitrogen, chloride, creatinine, globulin, glucose, gamma glutamyl transferase, iron, osmolality, phosphorus, sodium, total bilirubin, total calcium, triglycerides, total protein, uric acid were adjusted. CHO, total cholesterol.

The association between triglycerides and total lumbar BMD was positive in all three regression models (Table 4): model 1 (β = 0.024, 95% *CI*: 0.017, 0.031); model II (β = 0.021, 95% *CI*: 0.015, 0.028); and model III (β = 0.021, 95% *CI*: 0.014, 0.028). Moreover, the trend remained significant among the different triglycerides level quartile groups (*p* for trend <0.001). In the subgroup analysis stratified by gender, we also observed a positive association between triglycerides and total lumbar BMD after controlling for potential confounding factors, with all *p*-values < 0.001.

**TABLE 4:** Associations between triglycerides (mmol/L) and total spine BMD (gm/cm^2^).

	Model I OR (95% CI) *P*	Model II OR (95% CI) *P*	Model III OR (95% CI) *P*
TG	0.024 (0.017, 0.031) <0.001	0.021 (0.015, 0.028) <0.001	0.021 (0.014, 0.028) <0.001
**TG (Quartile)**			
Q1	Reference	Reference	Reference
Q2	0.030 (0.010, 0.051) 0.004	0.037 (0.018, 0.056) <0.001	0.031 (0.012, 0.050) 0.001
Q3	0.036 (0.016, 0.057) <0.001	0.049 (0.029, 0.068) <0.001	0.041 (0.022, 0.061) <0.001
Q4	0.085 (0.064, 0.106) <0.001	0.088 (0.068, 0.108) <0.001	0.082 (0.061, 0.103) <0.001
*P* for trend	<0.001	<0.001	<0.001
**Gender**			
Males	0.014 (0.006, 0.021) <0.001	0.016 (0.009, 0.024) <0.001	0.014 (0.006, 0.022) 0.001
Females	0.033 (0.019, 0.046) <0.001	0.042 (0.029, 0.055) <0.001	0.035 (0.021, 0.048) <0.001

Model I: None covariates were adjusted; Model II: gender (not adjusted for in the subgroup analyses), age and race were adjusted; Model III: gender (not adjusted for in the subgroup analyses), age, race, marital status, alanine aminotransferase, albumin, alkaline phosphatase, bicarbonate, blood urea nitrogen, chloride, creatinine, globulin, glucose, gamma glutamyl transferase, iron, osmolality, phosphorus, sodium, total bilirubin, total calcium, cholesterol, total protein, uric acid were adjusted. TG, Triglycerides.

**FIGURE 2:**
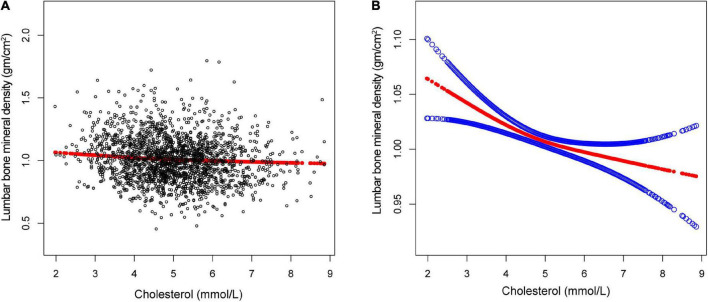
The association between total cholesterol and lumbar bone mineral density (BMD). **(A)** Each black point represents a single participant cholesterol sample. **(B)** Solid red line represents the smooth curve fit between variables. Blue bands represent the 95% of confidence interval from the fit. Gender, age, race, marital status, alanine aminotransferase, albumin, alkaline phosphatase, bicarbonate, blood urea nitrogen, chloride, creatinine, globulin, glucose, gamma glutamyl transferase, iron, osmolality, phosphorus, sodium, total bilirubin, total calcium, triglycerides, total protein, and uric acid were adjusted.

**FIGURE 3:**
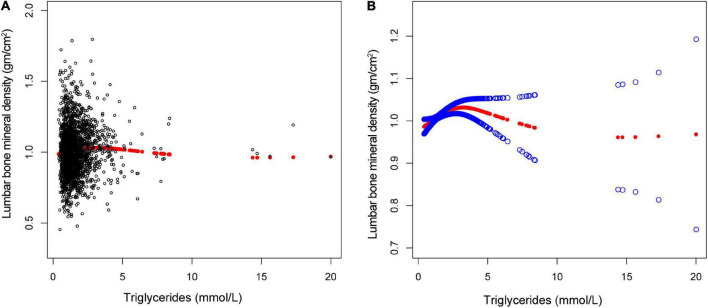
The association between triglycerides and lumbar bone mineral density (BMD). **(A)** Each black point represents a single participant triglycerides sample. **(B)** Solid red line represents the smooth curve fit between variables. Blue bands represent the 95% of confidence interval from the fit. Gender, age, race, marital status, alanine aminotransferase, albumin, alkaline phosphatase, bicarbonate, blood urea nitrogen, chloride, creatinine, globulin, glucose, gamma glutamyl transferase, iron, osmolality, phosphorus, sodium, total bilirubin, total calcium, cholesterol, total protein, and uric acid were adjusted.

The non-linear relationship between total cholesterol, triglycerides, and total lumbar BMD is shown in Figures 2, 3, respectively. Using a two-piecewise linear regression model, the point of inflection in the *U*-shaped association was at a level of 6.077 mol/L for total cholesterol and 2.597 mol/L for triglycerides. On both sides of the inflection point, there were higher cholesterol levels and lower lumbar BMD (Table 5). To the left of the inflection point, there was a higher triglycerides level and lumbar BMD. To the right of the inflection point, there was a higher triglycerides level and lower lumbar BMD, but the difference was not statistically significant (Table 6).

**TABLE 5:** Threshold effect analysis of total cholesterol on lumbar BMD using the two-piecewise linear regression model.

Lumbar BMD	Adjusted β (95% CI) *P*
Fitting by the standard linear model	−0.022 (−0.028, −0.015) <0.0001
**Fitting by the two-piecewise linear model**	
Inflection point	6.077
Total cholesterol <6.077 (mmol/L)	−0.018 (−0.027, −0.010) <0.0001
Total cholesterol >6.077 (mmol/L)	−0.033 (−0.055, −0.012) 0.0019
Log likelihood ratio	0.243

Gender, age, race, marital status, alanine aminotransferase, albumin, alkaline phosphatase, bicarbonate, blood urea nitrogen, chloride, creatinine, globulin, glucose, gamma glutamyl transferase, iron, osmolality, phosphorus, sodium, total bilirubin, total calcium, triglycerides, total protein, uric acid were adjusted.

**TABLE 6:** Threshold effect analysis of triglycerides on lumbar BMD using the two-piecewise linear regression model.

Lumbar BMD	Adjusted β (95% CI) *P*
Fitting by the standard linear model	0.020 (0.013, 0.027) <0.0001
**Fitting by the two-piecewise linear model**	
Inflection point	2.597
Total cholesterol <2.597 (mmol/L)	0.045 (0.033, 0.058) <0.0001
Total cholesterol >2.597 (mmol/L)	0.001 (−0.010, 0.011) 0.8834
Log likelihood ratio	<0.001

Gender, age, race, marital status, alanine aminotransferase, albumin, alkaline phosphatase, bicarbonate, blood urea nitrogen, chloride, creatinine, globulin, glucose, gamma glutamyl transferase, iron, osmolality, phosphorus, sodium, total bilirubin, total calcium, cholesterol, total protein, uric acid were adjusted.

## Discussion

In this current study, we used the lasted representative data of NHANES (2017–2020) to evaluate the associations between total cholesterol, triglycerides, and total lumbar BMD in aged 50 years and over. The results revealed a negative association between total cholesterol and total lumbar BMD and a positive association between triglycerides and total lumbar BMD. Specifically, we identified a *U*-shaped association between cholesterol and total lumbar BMD, with an inverted *U*-shaped association between triglycerides and total lumbar BMD.

Abnormal cholesterol metabolism has been associated with several metabolic diseases. Numerous studies investigated the association between cholesterol and BMD in the general population, but conclusions were inconsistent. In most studies, a higher level of cholesterol was found to be associated with an increased risk of osteoporosis or a lower BMD (5–8). A non-association between cholesterol and BMD was also reported (9–11). Our findings that higher total cholesterol levels are associated with lower spine BMD do support the most previous research studies. However, in the subgroup analysis stratified by quartile level of total cholesterol, the negative association between cholesterol and lumbar BMD remained only in Q3 and Q4 subgroups. In the subgroup analysis stratified by gender, this negative association remained in the women group. These results suggest that different subgroups of the same variable may also affect this association. The mechanism of negative associations between cholesterol and osteoporosis is still unclear. The oxidized lipid could be one of the reasons to cause osteoporosis, by inhibiting osteoblast differentiation and reducing bone mineralization (12, 13). Other potential mechanisms may involve bone metabolic abnormalities due to inflammatory regulators and flow-limiting atherosclerotic hypoperfusion (14, 15).

Hypertriglyceridemia is one of the most common lipid abnormalities. Many diseases caused by hypertriglyceridemia have been identified. The links between triglyceride and BMD were widely studied in the general population with different conclusions. Multiple evidence supported that increased triglyceride level was significantly associated with a decreased BMD or osteoporosis (16). Consistent with the above, a positive association between them was also observed (17). In order to clarify the relationship between triglyceride and total spine BMD, our present study showed a positive association between them. In the subgroup analysis stratified by quartile level of triglycerides or by gender, this positive correlation maintains consistency. Our findings also suggested that there is an inverted *U*-shaped relationship between triglyceride and BMD by further threshold effect analysis. When a triglyceride is less than 2.597 (mmol/L), the relationship between triglyceride levels and lumbar BMD is positively correlated. When a triglyceride is greater than 2.597 (mmol/L), it seemed that an increased triglyceride level is associated with a decreased BMD, but the difference was not statistically significant. This may imply that triglyceride has a dose-dependent effect on total lumbar BMD. A normal or moderately high level of triglyceride plays a beneficial role on bone, while a very high level of triglyceride may be unrelated to bone health. Similar to our finding, the dose–response relationship of triglyceride to BMD also has been reported in previous studies (18).

Our study has several strengths compared to the previous studies. First, we used the lasted data from NHANES between 2017 and March 2020 pre-pandemic in aged 50 years and over. Second, our results demonstrated a positive association between triglyceride and total lumbar BMD with a point of inflection at 2.597 mmol/L. The inverted *U*-shaped association between triglycerides and total lumbar BMD was not revealed in the previous studies. The relationships between cholesterol, triglycerides, and BMD have been conflicting. The possible reasons for these differences may be associated with heterogeneity in sample size, participant type, and covariate correction. There are several limitations to this study. First, the NHANES database is a cross-sectional survey, thus temporality and causation cannot be inferred from this study. More fundamental mechanistic research and large sample prospective studies are needed to understand the particular mechanism of them. Second, data collection for the NHANES 2019–2020 cycle was not completed due to the coronavirus disease 2019 pandemic. Last, other possible covariates, such as therapeutic intervention, autoimmune, and other conditions, did not take into account. Different covariates may influence the links between cholesterol, triglycerides, and lumbar BMD. High-quality studies on the relationship between cholesterol, triglycerides, and total lumbar BMD are required to confirm or oppose our findings.

## Conclusion

In people aged 50 years and over, our research discovered a negative association between total cholesterol and total lumbar BMD and a positive association between triglycerides and total lumbar BMD. There was an inverted *U*-shaped association between triglycerides and total lumbar BMD with a point of inflection at 2.597 mmol/L.

## Data availability statement

The datasets presented in this study can be found in online repositories. The names of the repository/repositories and accession number(s) can be found in the article/Supplementary material.

## Ethics statement

Written informed consent was obtained from the individual(s) for the publication of any potentially identifiable images or data included in this article.

## Author contributions

PW performed and interpreted statistics and also drafted the initial manuscript. CC and CS were responsible for data collection and statistical analysis. JJ and YW supported the manuscript writing. WM contributed to the study design for the whole research and revised the manuscript. All authors read and approved the final manuscript.

## Footnotes

1
www.cdc.gov/nchs/nhanes/
