# Supplementary figures and images for: High cholesterol and low triglycerides are associated with total lumbar bone mineral density among adults aged 50 years and over: The NHANES 2017–2020
Source: Front Med (Lausanne). 2022 Aug 8;9:923730. doi: 10.3389/fmed.2022.923730 (PMC9393595; doi:10.3389/fmed.2022.923730)

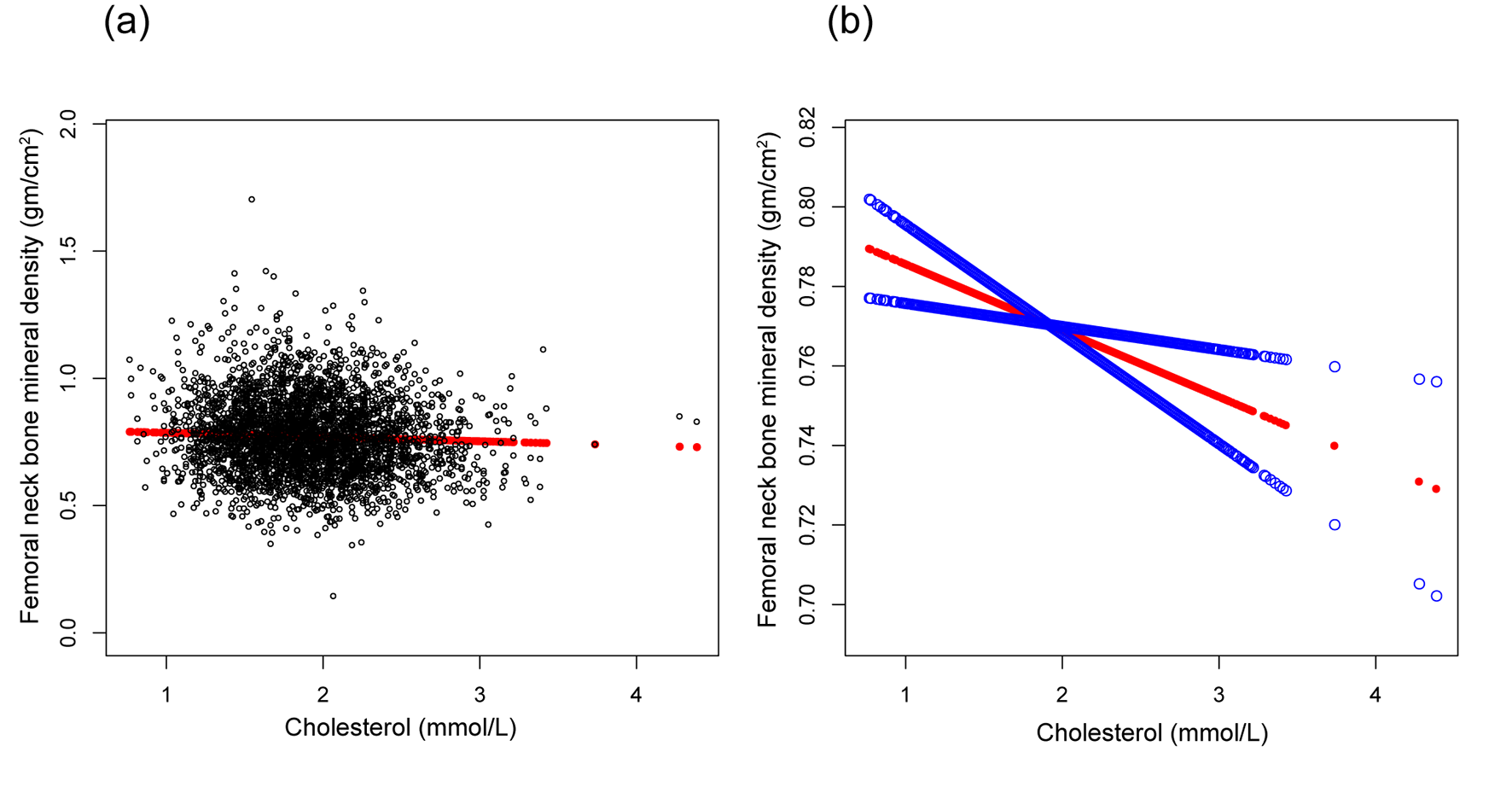

Supplement: Supplementary file 1 [file Image_1.TIF]

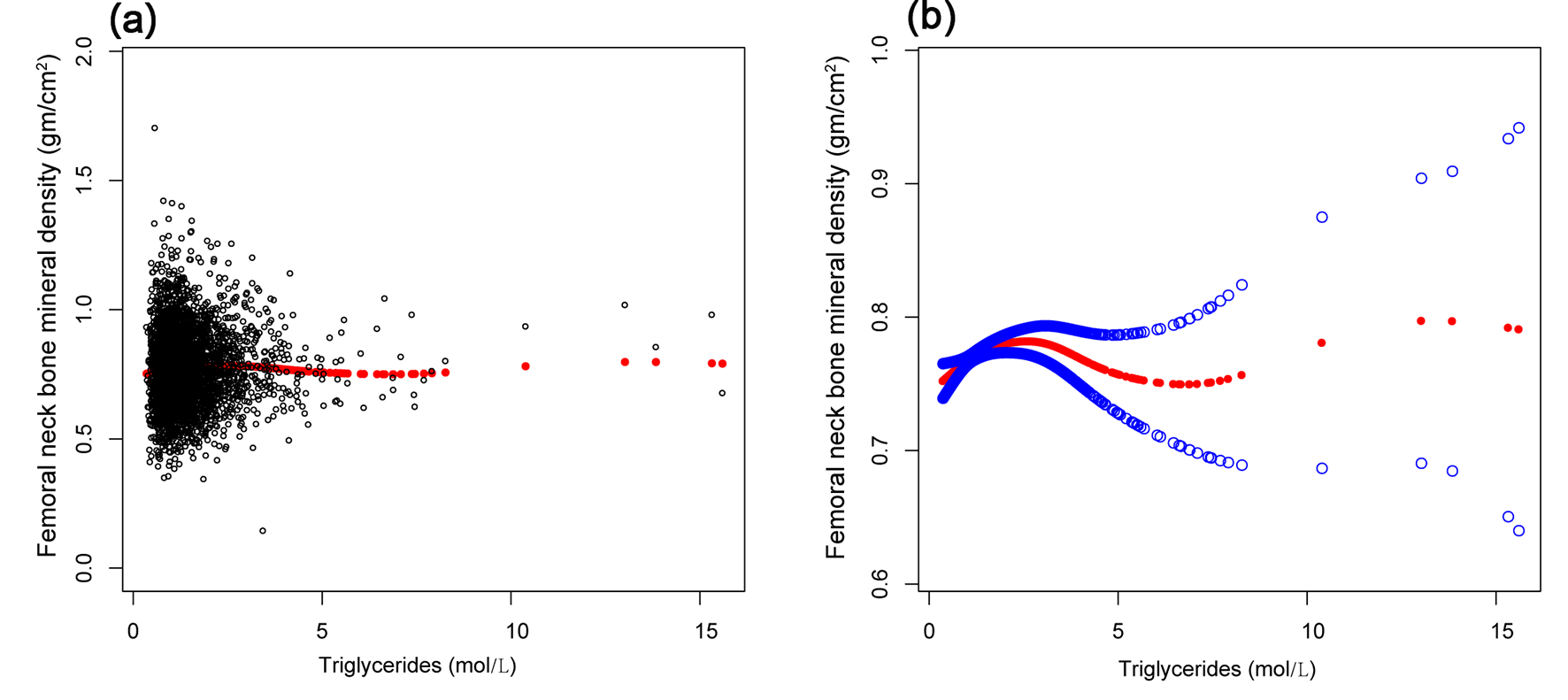

Supplement: Supplementary file 2 [file Image_2.TIF]
